# Impact of environmental factors on heat-associated mortalities in an urban desert region

**DOI:** 10.1007/s00484-022-02346-7

**Published:** 2022-09-10

**Authors:** Rachel A. Braun, David M. Hondula, Matthew P. Fraser

**Affiliations:** 1 Healthy Urban Environments Initiative, Global Institute of Sustainability and Innovation, Arizona State University, Tempe, AZ USA; 2 School of Geographical Sciences and Urban Planning, Arizona State University, Tempe, AZ USA; 3 School of Sustainable Engineering and the Built Environment, Arizona State University, Tempe, AZ USA

**Keywords:** Extreme heat, Air pollution, Mortality, Heat warning

## Abstract

The troubling trend of rising heat-associated mortalities in an urban desert region (Maricopa County, AZ, USA) has motivated us to explore the extent to which environmental factors may contribute to increased heat-health risks. Summertime data from 2010 to 2019 were used to construct a suite of models for daily heat-associated mortalities. The best-performing full model included the following predictors, ordered from strongest to weakest influence: daily average air temperature, average of previous 5 days daily average air temperature, year, day of year, average of previous 5 days daily average dew point temperature, average of previous 5 days daily average PM_2.5_, and daily average PM_10_. This full model exhibited a 5.39% reduction in mean absolute error in daily heat-associated mortalities as compared to the best-performing model that included only air temperature as an environmental predictor. The extent to which issued and modeled excessive heat warnings (from both the temperature only and full models) corresponded with heat-associated mortalities was also examined. Model hindcasts for 2020 and 2021 showed that the models were able to capture the high number of heat-associated mortalities in 2020, but greatly undercounted the highest yet observed number of heat-associated mortalities in 2021. Results from this study lend insights into environmental factors corresponding to an increased number of heat-associated mortalities and can be used for informing strategies towards reducing heat-health risks. However, as the best-performing model was unable to fully capture the observed number of heat-associated mortalities, continued scrutiny of both environmental and non-environmental factors affecting these observations is needed.

**Supplementary Information:**

The online version contains supplementary material available at 10.1007/s00484-022-02346-7.

## Introduction

As urban populations grow worldwide, human exposure to environmental hazards connected with cities will also increase. Among these hazards are both extreme heat, amplified in urban areas by the urban heat island effect, and poor air quality. While long-term trends in mortalities due to extreme heat across the USA have been decreasing nationally, this trend is not consistent in all regions (Sheridan et al. 2021). The persistence of extreme heat as a public health challenge motivates additional efforts to develop and improve preparedness and response activities.

Numerous studies have examined the relationships between temperature and mortalities. These works have sought to identify the types of temperature measurements (e.g., daily maximum, daily minimum, and daily average) that are the best predictors of mortalities (Barnett et al. 2010; Davis et al. 2016) and other environmental factors that influence these relationships, such as humidity and air quality (Chen et al. 2015). The potential for combined impacts of temperature and air pollution on mortality has been studied in locations throughout the world, including in Europe (Katsouyanni et al. 1993; Sartor et al. 1997; Stedman 2004; Filleul et al. 2006; Keatinge and Donaldson 2006; Stafoggia et al. 2008; Burkart et al. 2013; Analitis et al. 2104, 2018; Shaposhnikov et al. 2014; Willers et al. 2016; Krug et al. 2019, 2020), North America (Rainham and Smoyer-Tomic 2003; Roberts 2004; Ren et al. 2008; Basu et al. 2008; Zanobetti and Schwartz 2008; Jhun et al. 2014), Asia (Qin et al. 2017), and Australia (Ren et al. 2006), yielding mixed results. Other studies have examined relationships between mortalities and various types of heat-health warning systems (Hajat et al. 2010; Zhang et al. 2012). However, most of these works have examined all-cause mortalities, nonaccidental mortalities, excess mortalities, and/or cause-specific mortalities, such as cardiovascular or respiratory causes, but not heat as a specific cause. A few studies have sought to examine heat and air pollution relationships specifically to heat-related morbidities and mortalities (e.g., Yip et al., 2008; Williams et al., 2012). However, as mortalities directly associated with extreme heat continue to be a problem, additional work is needed to understand environmental factors that impact these cause-specific mortalities.

An ideal location for studying these issues and developing effective solutions is Maricopa County, AZ, USA, located in the Sonoran Desert and home to the Phoenix Metropolitan Area. Research into extreme heat and the urban heat island effect has been the subject of numerous studies in this region, especially when compared to other major urban areas in the USA (e.g., Chow et al. 2012a). Furthermore, the urbanized portions of Maricopa County are currently in non-attainment of the Environmental Protection Agency’s (EPA) National Ambient Air Quality Standards (NAAQS) for ozone (O_3_) and particulate matter with diameter less than 10 µm (PM_10_) (Environmental Protection Agency Green Book, 2021). Despite the desert environment and “heat-adapted” population, Maricopa County still accounts for a disproportionate number of heat-associated mortalities in the USA (Iverson et al. 2020).

The Maricopa County Department of Public Health (MCDPH) implemented a heat-associated mortality monitoring program in 2006 following an exceptionally hot summer in 2005 that saw an increase in heat-associated mortalities. Since that time, the annual number of heat-associated mortalities has grown from 85 in 2006 to 197 in 2019 (Maricopa County Department of Public Health 2019). Results from 2020 and 2021 indicate that these were the deadliest summers yet, with a total of 323 heat-associated mortalities in Maricopa County in 2020 and 329 in 2021 based on a preliminary report (Maricopa County Department of Public Health 2022). Projections of future scenarios (year 2050) based on varying amounts of urbanization and adoption of reflective roofs for the region have yielded a wide range of values for the expected number of heat-related mortalities (Hondula et al. 2014).

One mechanism for warning the public about risks from extreme heat in the USA is the issuance of “excessive heat warnings” by the National Weather Service (NWS). Quoting directly from the NWS Phoenix Weather Forecast Office Heat Safety webpage, “alerts are intended to raise awareness and prevent heat illness and death from occurring and mitigate financial impact” (https://www.weather.gov/psr/HeatSafety). Therefore, while prevention of heat-related mortalities is not the only the goal of these alerts, it is an important one nonetheless and perhaps one of the best reminders for the public about the dangers of extreme heat. However, the extent to which these reminders correspond to actual increased risk for heat-related mortalities in the region, which may depend not only on extreme heat for a given day but also heat from previous days and other environmental variables such as air pollution, is unknown.

This work seeks to address the following two questions: (1) does the inclusion of additional environmental factors beyond air temperature lead to better modeling of heat-associated mortalities and (2) how well do issued excessive heat warnings and modeled heat warnings correspond with heat-associated mortalities? As 2020 and 2021 were the deadliest summers yet in the region with respect to heat-associated mortalities, we apply the results from these two guiding questions to data from these summers. Ultimately, better understanding of conditions leading to increased risks for heat-associated mortalities can be used to develop strategies for combating the troubling trends in growing heat-associated mortalities in the region and enhance heat resilience and preparedness activities in urban areas where heat is a health challenge.

## Methods

### Study location

**Fig. 1:**
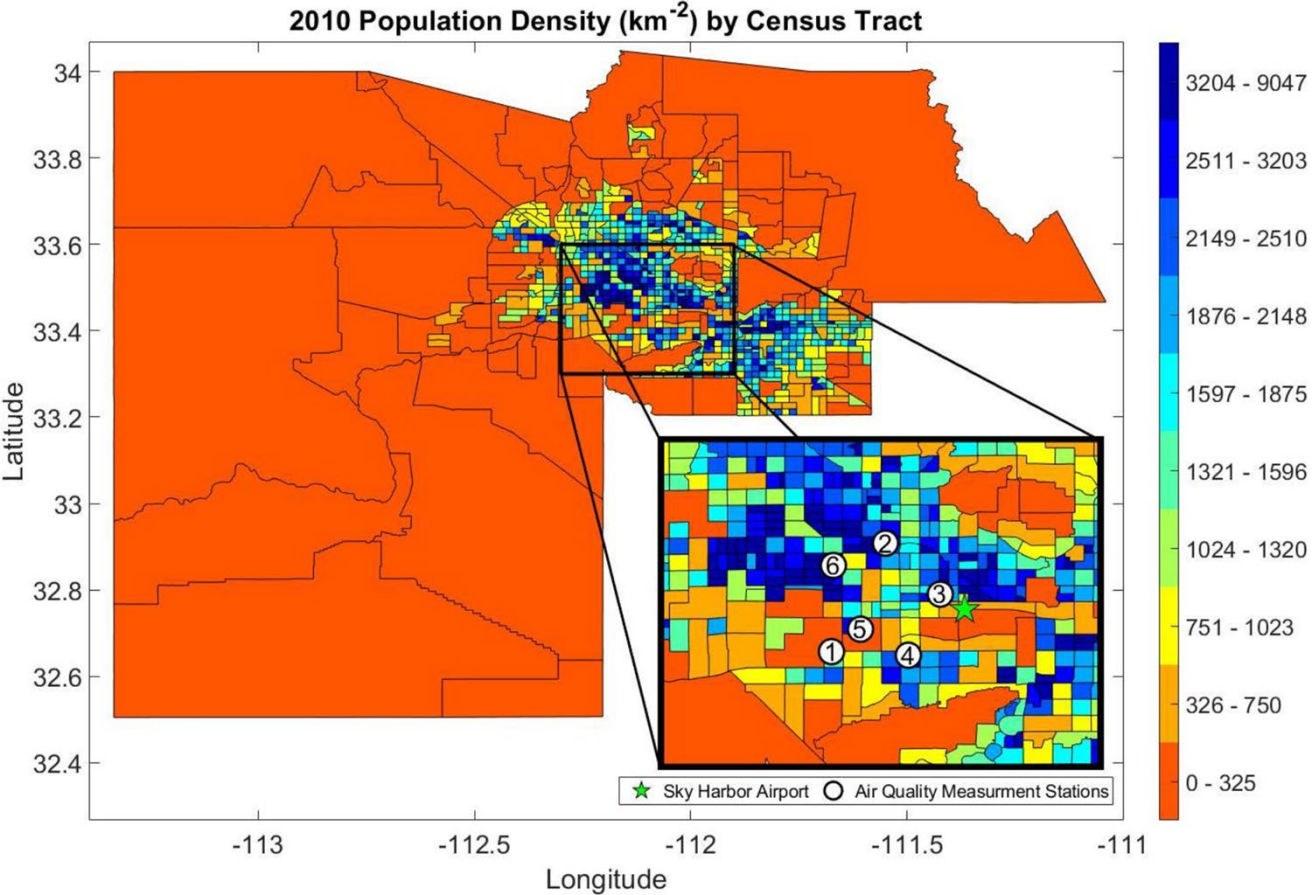
Map of Maricopa County with 2010 population density by census tract. Note that the color scale represents population density percentiles split into tenths for the region. The inset shows the locations of the centrally located measurement site for temperature (Sky Harbor Airport, green star) and air quality measurement stations. Sites 1–2 measured PM_10_ only, site 3 measured O_3_ and PM_10_, site 4 measured O_3_ and PM_2.5_, site 5 measured PM_2.5_ and PM_10_, and site 6 had measurements for all air pollutants (O_3_, PM_2.5_, and PM_10_)

Maricopa County is the most populous county in the state of Arizona and the fourth most populous county in the USA, with a population as of 1 July 2019 of approximately 4,485,000 (U.S. Census Bureau, Quick Facts). The county encompasses a wide variety of land use types, with large areas of both urban (i.e., the Phoenix Metropolitan area) and rural land. Figure 1 shows the 2010 population density obtained from the U.S. Census Bureau 2010 Census by census tract for Maricopa County.

### Data sources

Heat-associated mortality data were obtained from the Maricopa County Department of Public Health (MCDPH) heat surveillance program. The term “heat-associated mortality” is used by the MCDPH to include both heat-caused deaths and heat-related deaths. Definitions for these terms and methodology for identifying heat-associated mortalities can be found in annual summary reports published by MCDPH (e.g., Maricopa County Department of Public Health 2019).

Meteorological data from the Phoenix Sky Harbor International Airport, which is centrally located near downtown Phoenix (Fig. 1), has served as the source of high-quality environmental data for other previous studies of heat impacts on health in the region (e.g., Yip et al. 2008; Davis et al. 2016; Petitti et al. 2016; Putnam et al. 2018). Dry bulb temperature (also referred to as air temperature in this study) and dew point temperature were obtained from the National Oceanic and Atmospheric Administration (NOAA) National Centers for Environmental Information (NCEI) data archive for the Phoenix Sky Harbor International Airport site for 2010–2019. For the summers of 2020 and 2021, air temperature and dew point temperature data from Phoenix Sky Harbor International Airport were obtained from the MesoWest database (Horel et al. 2002).

A previous study examining the impacts of various environmental parameters, including air pollution, on heat-associated mortalities from 2000 to 2005 in Maricopa County used air pollution measurements from a single monitoring site in Phoenix (Yip et al. 2008). However, in this analysis, several stations near downtown Phoenix with available long-term data were used to find average concentrations for various air pollutants in the central urban area. Daily summaries for PM_2.5_ (24 h average), PM_10_ (24 h average), and O_3_ (maximum 8 h average) were obtained from the EPA Air Quality System (AQS). Locations for all air pollution measurement stations included for these average concentrations are displayed in Fig. 1.

Finally, historical data for “excessive heat warnings” in the Phoenix area issued by the National Weather Service (NWS) were obtained from the NWS Phoenix Weather Forecast Office Heat Safety webpage (https://www.weather.gov/psr/HeatSafety). Historical air quality warnings were obtained from the Maricopa County Air Quality Department webpage for “Air Quality Status and Monitoring” (https://www.maricopa.gov/1643/Air-Quality-Status-and-Monitoring).

### Model formula

Data from May to September, 2010–2019, were used to formulate a model for daily heat-associated mortalities in Maricopa County using both day-of and lagged responses from environmental variables. Lagged variables were specified as either the previous day measurement (1 day lag) or average of the previous *x* number of days, where *x* ranged from 2 to 5. Days with incomplete environmental data were excluded from analysis and the missing data were excluded from calculations of lagged environmental data for subsequent days for any lags greater than 1 day. Excluding days with incomplete environmental data resulted in 1523 days with a total of 1184 heat-associated mortalities used for the model formulation. In order to directly compare model coefficients, all variables were standardized according to the following formula:

1 $${X}_{\mathrm{standard}}=(Xi-{X}_{\mathrm{min}})/({X}_{\mathrm{max}}-{X}_{\mathrm{min}})$$

where *X*_standard_ is the unitless standardized version of the data point *X*_*i*_, *X*_min_ is the minimum value for the variable from May to September, 2010–2019, and *X*_max_ is the maximum value for the same time frame. Furthermore, the day of year variable was defined as days since April 30th to avoid discrepancies during leap years. Daily heat-associated mortalities were fitted using a general linearized model utilizing a Poisson distribution. The formula for the resulting model is as follows:

2 $${\mathrm{log}}_{e}(Y)={a}_{0}+{a}_{1}{X}_{\mathrm{standard},1}+{a}_{2}{X}_{\mathrm{standard},2}+\dots +{a}_{n}{X}_{\mathrm{standard},n}$$

where *Y* is the number of heat-associated mortalities, *α*_0_ is the model intercept, *X*_standard,*i*_ are the potential predictors, and *α*_*i*>0_ are the estimated coefficients for each predictor. For this study, the model was constrained to using linear terms only. Note that for all subsequent discussions, the daily heat-associated mortalities output by the model were rounded to the nearest whole number. Model performance was evaluated using mean absolute error (MAE) defined as average absolute difference between the number of observed and predicted daily heat-associated mortalities. The analysis was conducted using the fitglm function in MATLAB version 2020a.

The testing of various model configurations and choice of predictors will be discussed in further detail in the “Results and discussion” section. Day of week was not considered a predictor given the fairly consistent distribution of heat-associated mortalities across days of the week (Figure S1). Furthermore, interactive effects between environmental parameters were not considered, although exploration of potential for these types of effects presents an interesting area for future research.

## Results and discussion

### Overview of monthly heat-associated mortalities and environmental conditions

**Fig. 2:**
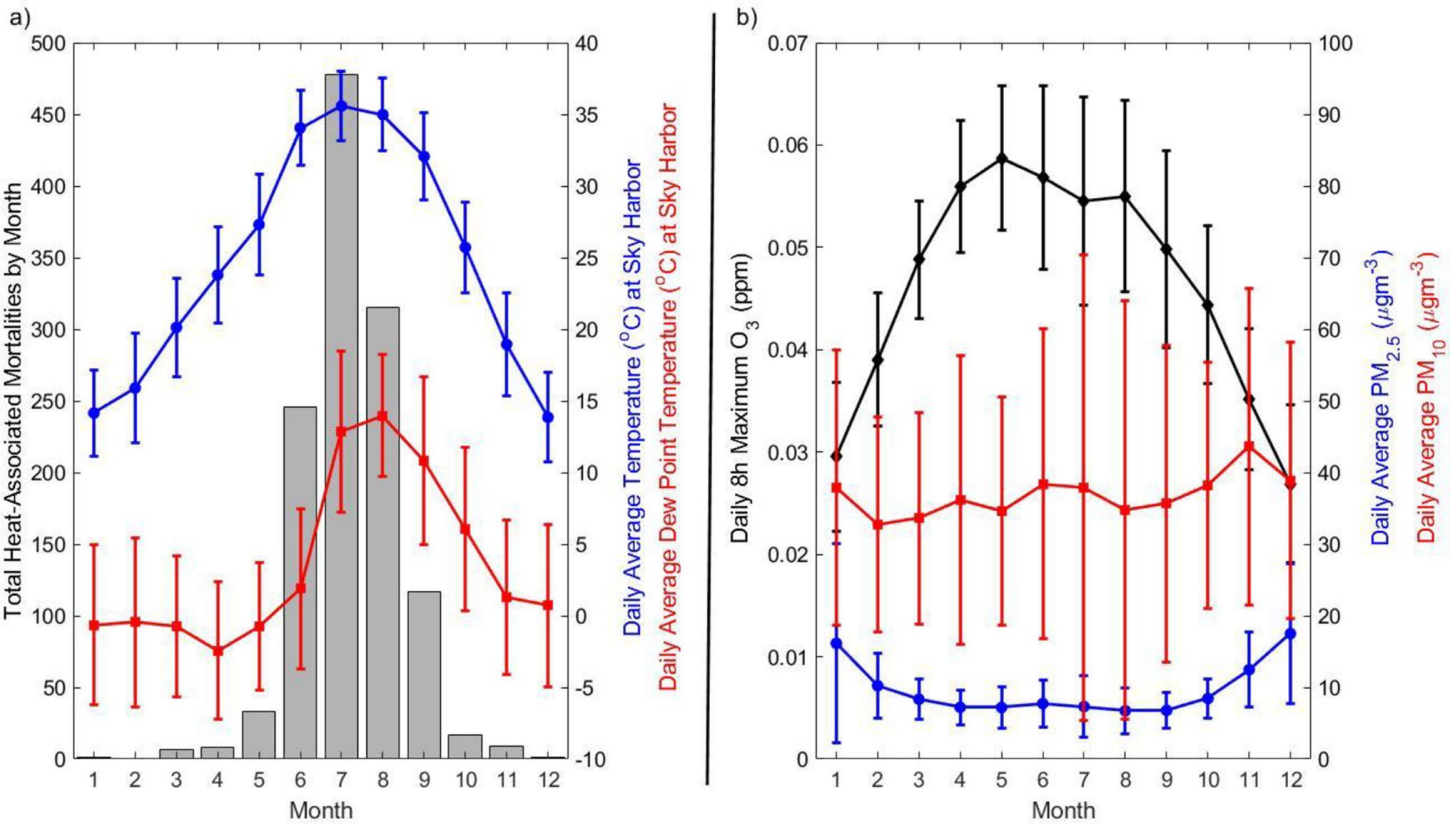
Summary of heat-associated mortalities and environmental conditions (panel **a**), and air pollutants (panel **b**) in Maricopa County by month for 2010–2019. The total heat-associated mortalities per month are shown as a bar graph, while all other variables are displayed as the mean ± one standard deviation

Heat-associated mortalities, air temperature, dew point temperature, and ozone all exhibited strong seasonal variability during the study period (Fig. 2). From 2010 to 2019, 1189 heat-associated mortalities occurred in the months of May–September, representing 96.5% of the 1232 total heat-associated mortalities for these years. Since a high fraction of the heat-associated mortalities occur in May–September, only data from these months were used in the model formulation.

Early summer (May–June) in Maricopa County is characterized by extremely hot and dry conditions. However, the arrival of the North American Monsoon around the beginning of July (Adams and Comrie 1997) brings increased moisture to the region, as evidenced by the large increase in average daily dew point temperature between June and July in Fig. 2a. For air pollutants (Fig. 2b), O_3_ concentrations are much higher during the summer months due to a variety of factors including increased solar radiation that drives photochemical reactions leading to ozone formation. In contrast, PM_2.5_ reaches a maximum during the winter months, due in part to increased emissions from wood burning and temperature inversions that can confine pollution to the surface level during winter months (Pope et al. 2017). Finally, PM_10_ is much more consistent year-round but shows large standard deviations for the daily averages within each month. Major sources of PM_10_ in the region include dust, which can become especially hazardous during the monsoon season when dust storms/haboobs typically form (Lader et al. 2016).

### Model parameterization

Several studies have previously examined environmental influences on heat-associated mortalities in Maricopa County. Yip et al. (2008) examined the relationships between meteorological variables and air pollution on heat-associated mortalities in Maricopa County from 2000 to 2005, while Putnam et al. (2018) examined the impact of temperature on heat-associated mortalities from 2006 to 2016. Harlan et al. (2014) examined the influences of apparent temperature, which is calculated using ambient temperature, dew point temperature, and wind speed, on both condition specific and all-cause mortalities in the region from 2000 to 2008. While each of these studies examined the impacts of various environmental factors, including extreme heat, on mortalities in Maricopa County, a new normal in the number of annual heat-associated mortalities is apparent starting in 2016, which is beyond the time frame of most of these previous studies. The present study uses data from 2010 to 2019 to examine the impacts of various environmental factors and characterize the change in model performance when adding complexity to the model through the inclusion of additional environmental factors beyond daily average air temperature.

Table 1 summarizes the steps taken to evaluate the performance of potential models with varying combinations of predictors. For each round of testing, all combinations of the listed variables were used to fit the model. For example, in round 1, the models tested included all seven possible permutations of the three variables (i.e., each variable alone, every combination of two variables, and a model with all three variables). For subsequent rounds, models were built in the same manner except without including multiple types of lags for the same variable (e.g., no models were tested that included both a 1 day lag and 2 day lag in air temperature).

**Table 1:** Summary of model formulations based on varying environmental predictors

Round of testing	Number of models tested	Potential predictors
1	7	Year
		Day of year
		Daily average air temperature
2	47	Predictors from round 1
		1–5 day lag daily average air temperature
3	575	Predictors from round 2
		1–5 day lag daily average dew point temperature
4	6911	Predictors from round 3
		Daily maximum 8 h average O_3_
		1–5 day lag daily maximum 8 h average O_3_
5	6911	Predictors from round 3
		Daily average PM_2.5_
		1–5 day lag of daily average PM_2.5_
6	6911	Predictors from round 3
		Daily average PM_10_
		1–5 day lag daily average PM_10_
7	82,943	Predictors from round 5
		Predictors from round 6

**Fig. 3:**
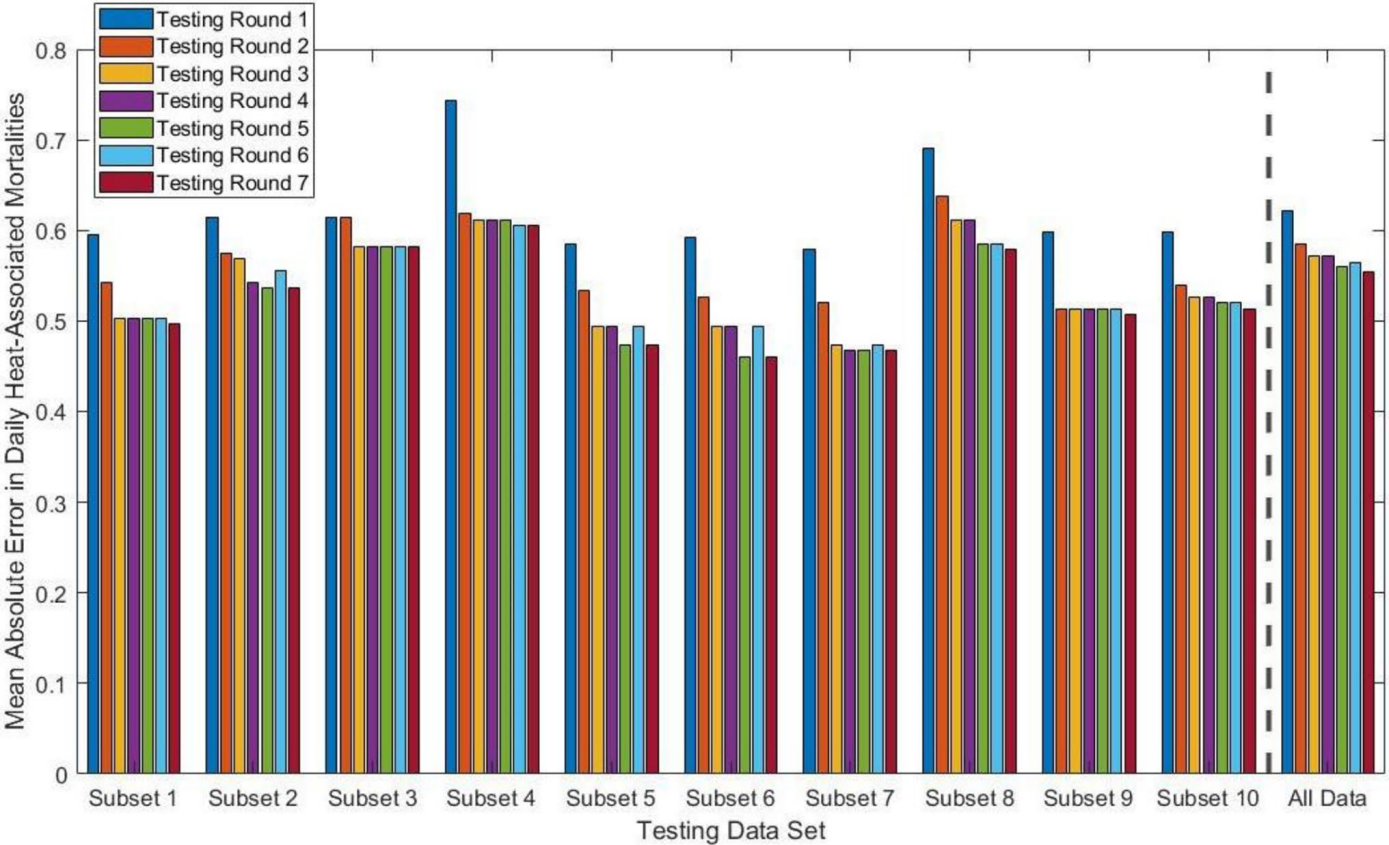
Mean absolute error (MAE) for daily heat-associated mortalities based on the best-performing model for each testing cycle. The testing rounds correspond to those listed in Table 1. For the data subsets listed, the best-performing model in each round was required to have *p*-value < 0.001 for each predictor variable and was selected based on the lowest MAE between the predicted and observed heat-associated mortalities when the fitted model was applied to the testing subset listed that was omitted from the model fitting. For the “All Data” category, the best-performing model in each testing round was selected by using the lowest MAE for the models built and tested using all of the data

For each round of testing, a data withhold and predict approach was used to test the robustness of the modeling approach and the influences of various environmental parameters. Each day used in the analysis was assigned to one of ten subsets of data. The rounds of testing summarized in Table 1 were used to fit the data in ten separate cycles of testing, with a subset of the data (i.e., 10%) being withheld each cycle and the model being subsequently fitted with the remaining 90% of the data. The fitted models were then used to predict the heat-associated mortalities in the omitted subset of data. All predictor variables in the best-performing model were required to be statistically significant with *p*-value < 0.001 and the best-performing model in each of these cycles was determined as the model which met the noted *p*-value condition and resulted in the lowest MAE in daily heat-associated mortalities between the observed heat-associated mortalities and the predictions based on the omitted subset of data, which were rounded to the nearest whole number for each day. In addition, a model built and tested using all of the data (i.e., no subset withheld) was also included. The results of the model cycling, and the comparison with the overall best-performing model based on the full dataset, are presented in Fig. 3. The formulations for the best-performing model in each testing cycle can be found in the Supplementary Information (Table S1). While the formulation for the best-performing model in each testing cycle differed, and differed from the overall best model, models including air quality information tended to perform better than those without. For all ten withhold and predict subsets, the worst performing model in each round of testing was that which only included day-of air temperature as a potential environmental predictor (round 1).

There were only 2 subsets withheld where the inclusion of O_3_ in round 4 improved model performance when compared to round 3 (i.e., the round with no air quality information but all other environmental parameters). Furthermore, for the model built and tested using all data, the inclusion of O_3_ did not improve model performance. However, the inclusion of PM data in round 5 (PM_2.5_) and round 6 (PM_10_) improved model performance as compared to round 3 for 6 and 4 of the withheld data subsets, respectively. In addition, both of these air quality measurements (PM_2.5_ and PM_10_) led to improvements in model performance for the model built and tested on the whole dataset. Therefore, round 7 included measures of both PM_2_._5_ and PM_10_, but not O_3._ While the data withhold and predict approach is useful for understanding potential improvements to model performance when including additional environmental parameters, the remainder of the discussion in this paper uses the best-performing models based on the full dataset.

Table 2 summarizes the coefficient estimates for the best-performing full model (round 7 in Table 1) based on the full dataset from 2010 to 2019, which included the environmental variables of air temperature, dew point temperature, PM_2.5_, and PM_10_. In addition, the collinearity of the variables was tested and the Pearson correlation coefficients (*R*) are presented in the Supplementary Information in Table S2. All variables had absolute values of R less than 0.5 except for three pairs of variables (daily average air temperature and average of previous 5 days daily average air temperature (*R* = 0.785), day of year and average of previous 5 days daily average dew point temperature (*R* = 0.703), and average of previous 5 days daily average air temperature and average of previous 5 days daily average dew point temperature (*R* = 0.514)). However, the analysis presented in Table 1 showed that adding each of these variables did result in improved model performance. The measures of PM were not strongly correlated (*R* = 0.267), perhaps due to the timing difference (i.e., day-of for PM_10_ vs. 5 day lag for PM_2.5_). Although not shown in Table S1 and not included in the best-performing model, day-of PM_2.5_ and PM_10_ exhibited a collinearity with *R* = 0.795, indicating that while the PM measurements are somewhat correlated, differing sources also most likely contribute to a weakened correlation between the two measures. The following section describes the choice of individual parameters and potential reasons for the observed model response.

**Table 2:** Summary of predictors and fitting parameters (coefficient estimate and standard error) for the best-performing full model formulated using Eq. 2. Predictors are organized by absolute value of the model coefficient from largest to smallest. Note that all predictors were statistically significant for *p* < 0.001. Variables were standardized using Eq. 1 to allow for comparisons between the model coefficients. The minimum and maximum values used in standardizing the variables via Eq. 1 are provided

Variable	Coefficient estimate (*α* in Eq. 2)	Standard error for coefficient	Minimum value (May–September, 2010–2019)	Maximum value (May–September, 2010–2019)
Intercept (*α*_0_ in Eq. 2)	-5.907	0.230	N/A	N/A
Daily average air temperature	3.451	0.307	18.33 °C	40.56 °C
Average of previous 5 days daily average air temperature	2.874	0.325	20.00 °C	39.56 °C
Year	1.141	0.104	2010	2019
Day of year (days since April 30th)	-0.998	0.202	1	153
Average of previous 5 days daily average dew point temperature	0.988	0.223	-14.00 °C	20.78 °C
Average of previous 5 days daily average PM_2.5_	0.822	0.229	3.08 µg m^−3^	21.53 µg m^−3^
Daily average PM_10_	0.803	0.241	6.25 µg m^−3^	299.60 µg m^−3^

High temperatures have been associated with increased risks for heat-related mortalities and hospitalizations in the region (Petitti et al. 2016). Furthermore, the urban heat island effect has played an exacerbating role in rising temperatures for the urban area, which is especially apparent at night. The use of daily average temperature, as opposed to daily maximum temperature, accounts for the sustained heat present at nighttime and has been used in other studies in the region (e.g., Putnam et al. 2018). Daily average temperature and average of the previous 5 days daily average temperature had the largest coefficients in the model (Table 2) and demonstrated the strongest impact on model performance out of all environmental variables tested. Dew point temperature was also included as a variable to account for the dramatic change in environmental conditions brought by the arrival of the North American monsoon typically in early July (Fig. 2a). The average of the previous 5 days daily average dew point temperature was used in the model and exhibited a smaller coefficient than that of air temperature. The effect of dew point temperature appeared to be slightly stronger than that of air pollution, although on the same order of magnitude.

As stated in the introduction, numerous studies around the world have examined the combined impacts of heat and air quality on mortalities. In the present study O_3_ was not found to be a strong predictor of daily heat-associated mortalities (Fig. 3), although previous work in the region has shown that O_3_ presents an increased relative risk for asthma hospitalizations (Mohamed et al. 2016). In contrast, both PM_2.5_ and PM_10_ were found to be better predictors for heat-associated mortality, albeit with a small effect. Previous studies in the region have shown associations between PM and negative health outcomes, including asthma (Dimitrova et al. 2012; Pope et al. 2017) and mortality (Mar et al. 2000). The best-performing model included both day-of PM_10_ and the average of the previous 5 days for PM_2.5_. While these two variables had lower coefficients as compared to air temperature, they nonetheless lead to an increase in model performance (round 7 in Table 1) as compared to the models without air pollution (round 3 in Table 1).

Another variable included in the model is day of year, specified as days since April 30th in order to normalize for leap year differences. This parameter was the only variable used in the model that resulted in a negative coefficient. This result appears to be in agreement with previous studies which have shown that early season heat waves pose greater risk for nonaccidental mortality than those later in the summer (e.g., Brooke Anderson and Bell 2011). We hypothesize that this variable and the negative coefficient could be due to a variety of reasons, including physiological and behavioral adaptations (e.g., Hondula et al. 2015b) by the local population over the course of the summer. Those most susceptible to heat-associated mortality (and morbidity) may have experiences early in the summer that lead them to seek additional help or develop coping mechanisms. Additional research should target the exact reasons for this observation, but the preliminary ideas presented here suggest that targeted early interventions both prior to and at the beginning of summer could be crucial in preventing adverse health effects.

Year is also included as a variable in the model due to a strongly apparent growing trend in heat-associated mortalities in the region. Unsurprisingly, year demonstrated a rather strong impact as evidenced by the high coefficient for this variable in Table 2. This variable could serve as a proxy for several effects, including increasing population and changing vulnerabilities associated with the current population. Population is rapidly growing in Maricopa County, with an estimated population growth from 2010 to 2019 of 17.5% (U.S. Census Bureau). In addition, the number of people experiencing unsheltered homelessness in Maricopa County has been growing, from 1,289 in 2015 to 3,767 in 2020 (Maricopa Association of Governments Point-In-Time Homeless Count). People experiencing unsheltered homelessness have been identified as a particularly vulnerable demographic for heat-associated mortalities in the region (Iverson et al. 2020).

### Comparison with temperature only model

As heat-associated mortalities would reasonably be expected to be most strongly related to measures of heat (i.e., air temperature), the performance of the “full model” from round 7 described in Table 2 is compared to that of the best model which only includes air temperature as an environmental parameter (subsequently referred to as the “temperature only” model). The characteristics of the best-performing temperature only model (from round 2 described in Table 1) are provided in Table 3. For this temperature only model, the strongest predictor is the average of the previous 5 days daily average air temperature, followed by the daily average air temperature.

**Table 3:** Summary of predictors for the best-performing model that included only air temperature as an environmental parameter (i.e., temperature only model). In the same way as Table 2, predictors are ordered by absolute value of the model coefficient from largest to smallest and all predictors were statistically significant for *p* < 0.001

Variable	Coefficient estimate (*α* in Eq. 2)	Standard error for coefficient
Intercept (*α*_0_ in Eq. 2)	-5.569	0.224
Average of previous 5 days daily average air temperature	3.560	0.313
Daily average air temperature	3.428	0.309
Year	0.962	0.093
Day of year (days since April 30th)	-0.604	0.150

**Fig. 4:**
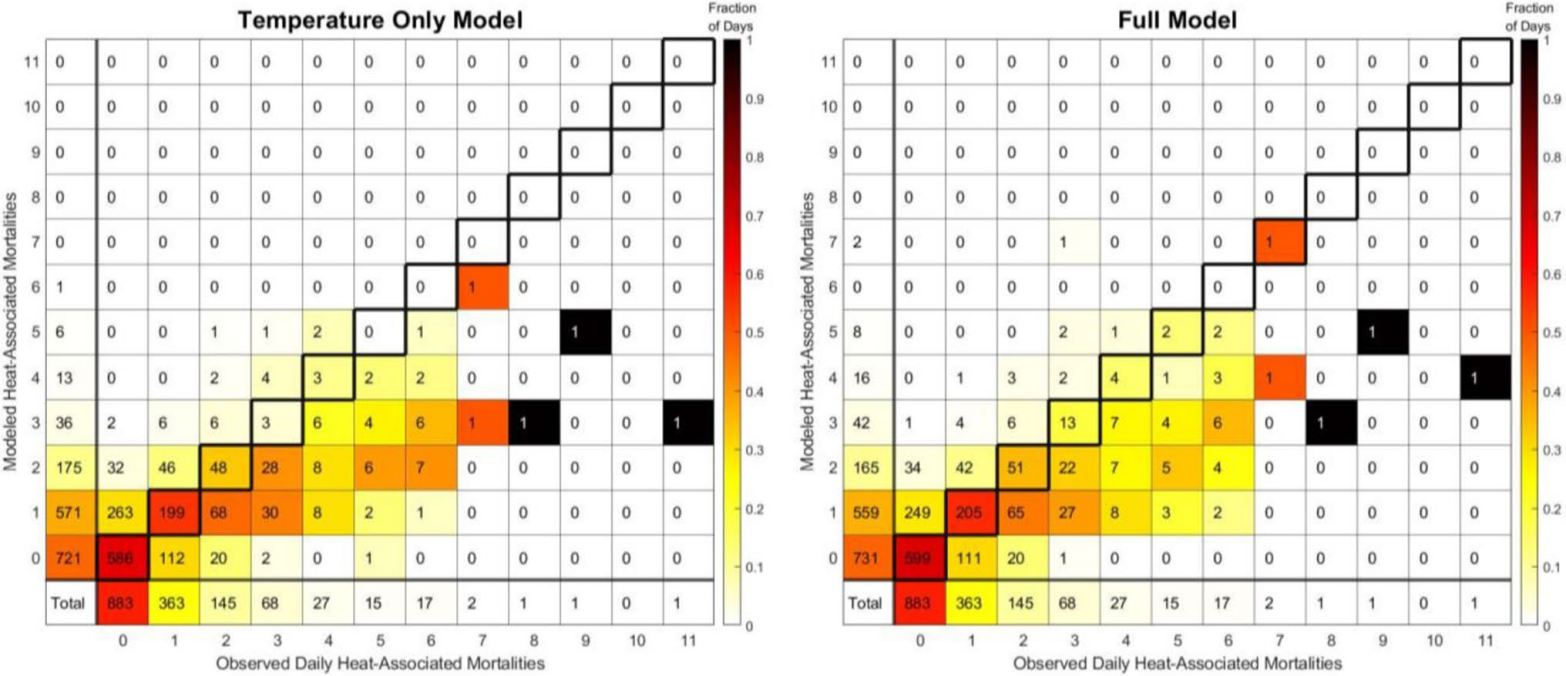
Days with the modeled and observed corresponding number of heat-associated mortalities for May–September, 2010–2019, for the temperature only model (left) and full model (right). For the “total” row and column, the color scale indicates the fraction of all days with either that predicted (first column) or observed (first row) number of heat-associated mortalities. For all other entries, the color scale indicates the fraction of days with each modeled number of heat-associated mortalities that correspond to a given number of observed daily heat-associated mortalities. In this way, the fraction of days (color scale) sums to one for each column (excluding the totals in the first row).The boxes outlined in thick black lines indicate the correct counts, with all areas above these boxes representing overcounts by the model and all areas beneath these boxes representing undercounts by the model

Overall, the MAE for the temperature only model was 0.585 and the MAE for the full model was 0.554, indicating a 5.39% reduction in MAE through the inclusion of additional environmental variables. The daily model performances, as compared to the actual number of observed heat-associated mortalities, are provided in Fig. 4. The full model produced accurate results on 57.45% of days, undercounts on 19.83% of days, and overcounts on 22.72% of days. In comparison, the temperature only model was less accurate overall (55.09% of days with correct counts), while 20.95% of days were undercounts and 23.07% of days were overcounts. Of particular interest, especially from a warning standpoint as will be discussed further in the next section, would be days where heat-associated mortalities occurred but model results indicated zero heat-associated mortalities. For these cases, the model results were similar between the temperature only model and full model, with the full model having 132 days meeting these criteria and the temperature only model with 135 cases.

### Model performance compared to warnings

Excessive heat warnings are issued each summer for the Phoenix area (i.e., urban Maricopa County) by the NWS. According to the NWS Phoenix Heat Safety webpage (https://www.weather.gov/psr/HeatSafety), these warnings are issued based on the level of risk determined by the NOAA/NWS HeatRisk product (https://www.wrh.noaa.gov/wrh/heatrisk/). The following section discusses warning days indicated by the models as compared to the actual historical heat warning days in the specific context of heat-associated mortalities; however, a few important caveats to the analysis should be noted. First, the following analysis is based retrospectively on observed data. In contrast, as warnings are issued in advance of events, they are based on forecasted data and are therefore inherently limited by the accuracy of forecasting. Second, issuance of heat warnings may induce behavioral changes in individuals and operation of additional services and resources (such as cooling centers) that may decrease risks for heat-associated mortalities. Therefore, the days with historical heat warnings may have lower numbers of heat-associated mortalities as compared to an alternative scenario where a warning was not issued on that particular day. Finally, this analysis is based solely on statistical outputs and does not consider risk communication strategies that may favor issuing warnings on days that do not fully meet the statistical thresholds for a warning but may nonetheless benefit from a heat warning based on other principles (e.g., a slightly cooler day sandwiched between heat warning days). Overall, the results from this analysis are not intended to serve as a comprehensive evaluation of the effectiveness of the current heat warning system in the region nor should they be interpreted as specific recommendations for warning system adjustments. While noting these conditions, the following analysis is still useful for identifying strengths and limitations of the current warning system as specifically related to the ability to capture regional heat-associated mortalities.

Although only 176 days (11.56% of the days examined) had excessive heat warnings, heat-associated mortalities on these days (336 heat-associated mortalities) accounted for 28.38% of all heat-associated mortalities examined. However, this still leaves 848 heat-associated mortalities (71.62%) occurring on days with no excessive heat warnings. Within this group, there were 85 days with no excessive heat warnings and 3 or more heat-associated mortalities, resulting in a total of 322 heat-associated mortalities (27.20% of all heat-associated mortalities examined). In addition, on 23.86% of the days with a heat warning issued, no heat-associated mortalities occurred.

To address these discrepancies, the temperature only and full models were used to identify days that would be prime candidates for issuing warnings regarding heat-associated mortalities. To identify these days, a threshold of two modeled daily heat-associated mortalities was chosen (i.e., any days with two or more modeled heat-associated mortalities would produce a warning). Because year is a variable in the model and in order to make a valid comparison across multiple summers for the sake of issuing warnings, year was held constant as the halfway point between the bounding model years of 2010 and 2019. If this step was not undertaken, the models indicate significantly more warning days in later years as opposed to earlier years in the case of similar environmental states. The number of days indicated for warnings is sensitive to the choice of the constant year in this analysis; if the year was held constant as 2010 there would be many less warning days, while the opposite would be true for year held constant as 2019. However, the choice of halfway for year seems to provide for a fair comparison since the resulting number of warning days for the models, discussed further below, is similar to the actual number of heat warning days.

Both models indicate an increase in the number of observed heat-associated mortalities captured by the updated set of warning dates, with 446 heat-associated mortalities (37.67% of heat-associated mortalities) on 195 days (12.80% of days) for the temperature only model and 439 heat-associated mortalities (37.08% of heat-associated mortalities) on 188 days (12.34% of days). Overall, this translates to an average of 2.29 observed heat-associated mortalities per warning day indicated by the temperature only model and 2.34 observed heat-associated mortalities per warning day indicated by the full model in comparison to 1.91 observed heat-associated mortalities per actual heat warning day. In addition, the number of days with no predicted warning but when three or more heat-associated mortalities occurred was 60 days for each model. Finally, there were no observed heat-associated mortalities on only 17.95% of days with warnings indicated by the temperature only model and 18.62% of days with warnings indicated by the full model.

**Fig. 5:**
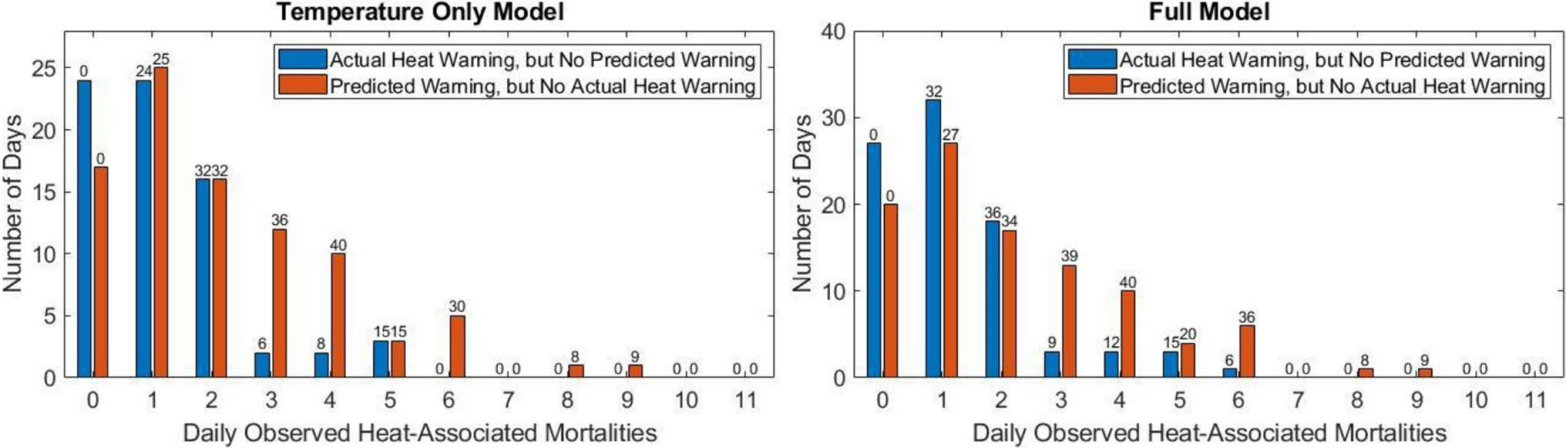
Scenarios in which there was a difference in warnings between actually issued warnings and model predicted warnings. Bars indicate the number of days falling into each category, while the numbers at the top of the bars indicate the number of heat-associated mortalities in the category. The first plot shows results from the temperature only model, while the second plot is for the full model

There were 105 days with both an actual heat warning and a predicted heat warning from the temperature only model; in the case of the full model, these overlapping days totaled 89. The distribution of overlapping days with and without warnings can be found in Figure S2 in the supplement. However, there were 71 days with an actual heat warning but no predicted warning from the temperature only model and 87 days with an actual heat warning but no predicted warning from the full model. For the inverse case (i.e., no actual heat warning but a warning was predicted by the model), the total number of days was 90 for the temperature only model and 99 from the full model. The results of these differences, including the number of heat-associated mortalities captured and missed in each scenario, are presented in Fig. 5. Both the temperature only model and full model identified for warnings many days with three or more heat-associated mortalities and no issued heat warning.

In addition to excessive heat warnings issued by the NWS, high pollution advisories (HPA) and health watches for air pollutants are issued by the Arizona Department of Environmental Quality for days with poor air quality in the region. However, as shown in Fig. 2b, O_3_ is of greater concern in the region during the summer months, while PM_2.5_ peaks during winter months. The historical warning data for air quality in the region reveals that while 315 either HPA or health watches for O_3_ were issued in May–September, 2013–2020, only 28 such warnings were issued for PM_10_ and just 2 for PM_2.5_ during this same time frame. This result is not unexpected given that the warnings are related to the National Ambient Air Quality Standards (NAAQS). For example, in the current study, our calculations for averages of PM_10_ and PM_2.5_ based on the monitors in Fig. 1 yielded only 16 days from May to September, 2010–2019, where the average PM_10_ exceeded the NAAQS 24-h PM_10_ standard of 150 µg m^−3^ and only 1 day where the average PM_2.5_ exceeded the NAAQS 24-h PM_2.5_ standard of 35 µg m^−3^_._ However, as shown in Fig. 3, inclusion of PM measurements in the model improved performance, while inclusion of O_3_ did not. Therefore, while the PM levels may tend to fall below the NAAQS standards during the summer in Maricopa County, negative health impacts due to the coupling of PM levels with other environmental factors, such as extreme heat, are plausible. Because there were so few PM pollution warnings issued during summer months (as expected based on the NAAQS), our analysis only focused on excessive heat warnings.

### Hindcasts for 2020 and 2021

The summer of 2020 broke numerous local climate records for extreme heat, including the records for average temperature in July and August in Phoenix (National Weather Service, 2020). In addition, there were 48 days with “excessive heat warnings” issued by the NWS (including 5 in April), compared to an annual maximum of 26 from 2010–2019. Furthermore, changes related to the COVID-19 pandemic, including closures of cooling centers and eviction moratoriums, likely impacted the number of heat-associated mortalities. In 2021, the overall summer was less hot than 2020, but the preliminary number of heat-associated mortalities was the highest of the last 20 years (Maricopa County Department of Public Health, 2022).

**Fig. 6:**
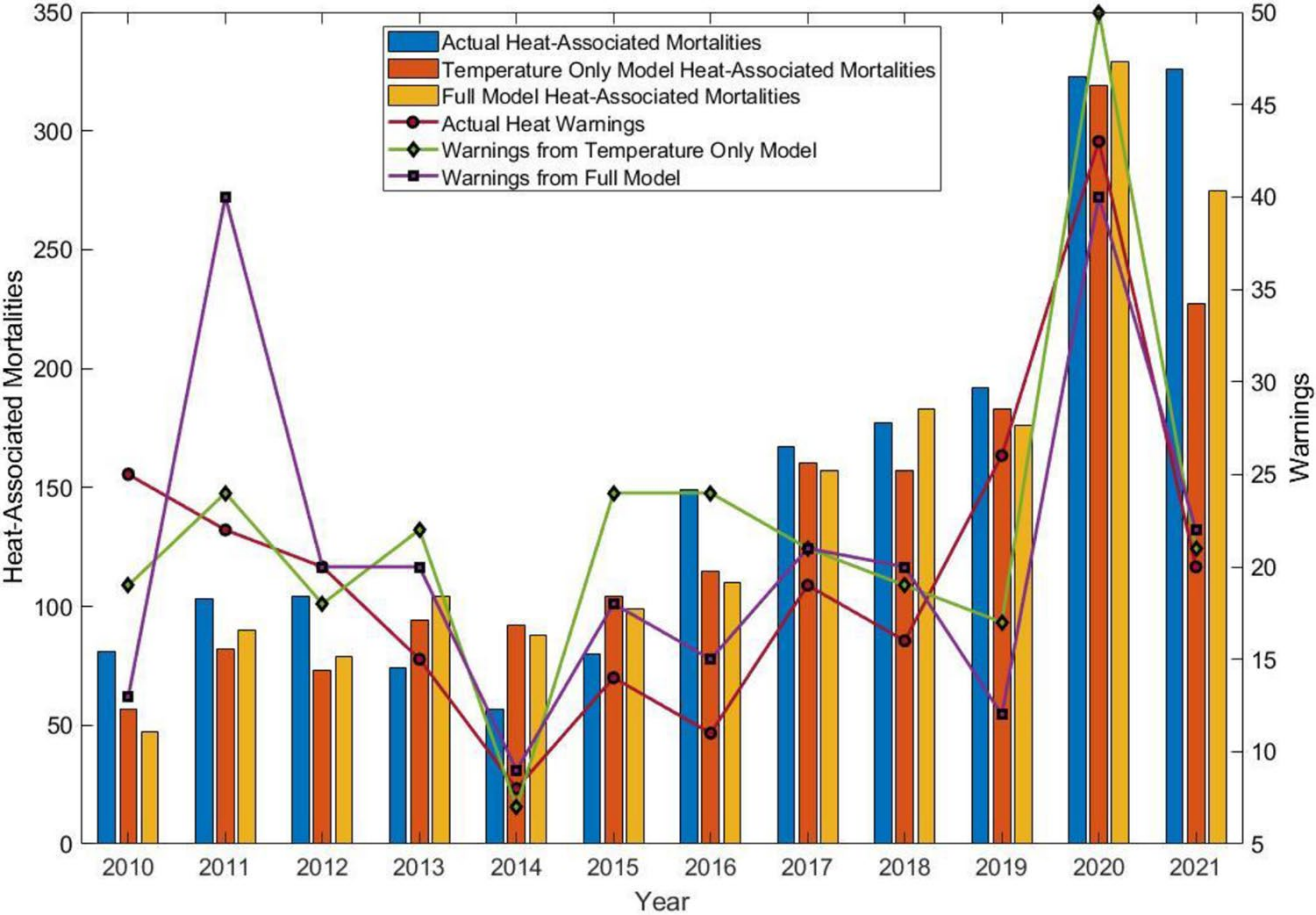
Annual heat-associated mortalities in Maricopa County from 2010 to 2021. Note that actual heat-associated mortalities in Maricopa County for 2020 include data from April to October; all other quantities shown are based on May–September. The model-predicted values for 2020 and 2021 are hindcast for May–September based on the model built using data from 2010 to 2019. Total days with excessive heat warnings are based on NWS issued warnings from May to September each year and model predictions for warnings are based on the methodology described in the “Model performance compared to warnings” section

A previous study examined the spike in heat-associated mortality that occurred in Maricopa County in 2016 (Putnam et al. 2018). Since that time, the “spike” in heat-associated mortalities in the county has become a disconcerting new normal, with the number of mortalities increasing each summer since 2015 (Fig. 6). The numbers of heat-associated mortalities in Maricopa County in 2020 and 2021 were the highest yet, with 323 heat-associated mortalities from April to October, 2020 (Maricopa County Department of Public Health, 2021), and 326 from May to September, 2021 (Maricopa County Department of Public Health, 2022). While this number of cases in 2020 includes the months of April and October, the MCDPH report indicates that approximately 97% of the heat-associated mortalities for the full summer of 2020 occurred in May–September. The model developed in this study (based on May–September, 2010–2019) was used to hindcast heat-associated mortalities in May–September, 2020 and 2021. As shown in Fig. 6, this exercise yielded similar hindcast values for May–September, 2020 for both the temperature only and full models. However, in 2021, the heat-associated mortalities hindcast by the full model and temperature only models were much lower than the observed number of heat-associated mortalities. In addition, the full model predicted 48 more heat-associated mortalities than the temperature only model; this was by far the largest yearly discrepancy between these two models, with the second largest discrepancy being 26 in 2018.

The number of heat warnings issued by year was also compared with the warnings that would result from the models using the methodology from the “Model performance compared to warnings” section. Although differences in the actual warning days were described in the “Model performance compared to warnings” section, the yearly totals for warnings tended to be somewhat similar between the three cases (i.e., actual and model indicated warnings), especially in 2021.

### Study limitations and implications for future research

While these results add to the growing body of literature examining environmental impacts on heat-associated mortalities, the current study has a few limitations in terms of the factors included in the analysis. One noted limitation of the current study is the lack of spatial information, which may be of particular relevance given that temperature and air quality can vary at small spatial scales. Research in the Phoenix Metropolitan area has shown that temperatures can vary at the neighborhood-level scale (Harlan et al. 2006; Ruddell et al. 2012). Furthermore, the wide variety of land use types across Maricopa County, coupled with the urban heat island effect, can present differences in temperature between the urban and rural areas. Given these challenges, it can be difficult to produce a singular temperature measurement representative of the whole study domain. Future research should analyze the extent to which the relationships examined in this work change based on considerations of spatial variability in temperature data. While the present study used air quality data from multiple sensors in the urban Phoenix area, identifying the extent to which air quality variability across the region impacts these relationships could also present an opportunity for further study.

As there is still error between the best-performing model and actual number of heat-associated mortalities observed, future research should also identify non-environmental factors, as well as additional environmental factors not included here, that may be driving this discrepancy. Previous research in the region has shown that people’s experiences with heat and thermal comfort can vary at the neighborhood scale (Harlan et al. 2006, 2013; Hayden et al. 2011). Vulnerability to extreme heat has been shown in the region to vary spatially (Hondula et al. 2015a) and among different demographics (Chow et al. 2012b; Chuang and Gober 2015; Chakalian et al. 2019). Furthermore, other previously identified risk factors in the region, such as homelessness and occupation (Petitti et al. 2013), were not included in this analysis. Therefore, while this study identifies environmental risk factors that can predict heat-associated mortalities, targeted interventions for preventing heat-related mortalities should also include guidance from previous works that have identified specifically vulnerable populations and areas. Proposed strategies include improving warning systems (Kalkstein and Sheridan 2007) and opening heat refuges/cooling centers in locations that are both accessible and serve populations with the highest needs (Eisenman et al. 2016; Berisha et al. 2017; Fraser et al. 2017, 2018). Engaging with local communities (Guardaro et al. 2020) and a variety of stakeholders (Guyer et al. 2019) will also be critical for addressing both short-term and long-term solutions for managing exposure to extreme heat and poor air quality.

In a study of elderly across the USA, Weinberger et al. (2021) did not find evidence of lower mortality on days with heat warnings; however, the authors did provide a diagram with hypothesized benefits of heat warnings for the sake of reducing heat-health impacts (Fig. 3 of Weinberger et al. 2021). As the results of this study suggest different days would be identified for warnings based on the models (which were built on observed data) as opposed to the actual warnings (based on forecasted data), the accuracy of forecasting both short-term and long-term temperature and air pollution conditions will be critical for developing more targeted warning systems.

## Conclusions

This study sought to answer two questions related to heat-associated mortalities in an urban-desert region (Maricopa County, AZ). The first question regarded the extent to which the inclusion of additional environmental factors beyond air temperature increased the performance of a model for heat-associated mortalities. The environmental factors tested as potential predictors included both day-of and lagged impacts of air temperature, dew point temperature, and air quality measurements for ozone, PM_2.5_, and PM_10_. Models were constructed using a data withhold and test approach using data from May to September, 2010–2019. An overall best-performing model showed that the inclusion of each additional parameter except O_3_ improved the performance of the model as compared to the model that only included air temperature as an environmental predictor. The best-performing model using only air temperature as an environmental factor, based on summertime data from 2010 to 2019, included as predictors, in order from strongest to weakest influence, average of previous 5 days daily average air temperature, daily average air temperature, year, and day of year. In the case of the full best-performing model, the predictors were daily average air temperature, average of previous 5 days daily average air temperature, year, day of year, average of previous 5 days daily average dew point temperature, average of previous 5 days 24-h average PM_2.5_ and 24-h average PM_10._ The MAE between the modeled and observed heat-associated mortalities was reduced from 0.585 for the best-performing temperature only model to 0.554 for the full best-performing model. However, as the average daily number of heat-associated mortalities in the region for this study period was 0.777, additional research will be required to identify both environmental and non-environmental factors that may be driving this discrepancy. This is especially needed given the high numbers of heat-associated mortalities in 2020 and 2021; while the temperature only and full models were able to capture the total number of heat-associated mortalities in 2020, both models exhibited large undercounts for the number of heat-associated mortalities in 2021.

The second question addressed by this study was the extent to which issued excessive heat warnings correspond to the observed daily heat-associated mortalities and whether models would indicate different days as optimal for warnings. For May–September, 2010–2019, there were an average of 1.91 heat-associated mortalities per day with an issued heat warning. Using a methodology for identifying a comparable total number of days to those with an issued heat warning, the temperature only model and full model were used to identify the best potential warning days based on modeled heat-associated mortalities. This process yielded a set of warning days with an average of 2.29 observed heat-associated mortalities per warning day indicated by the temperature only model and 2.34 observed heat-associated mortalities per warning day indicated by the full model.

In summary, we found that environmental factors beyond air temperature have a small but measurable influence on daily heat-associated mortalities in Maricopa County, AZ. Efforts to design, implement, improve, and evaluate regional preparedness and resilience strategies for extreme heat may be able to be improved by accounting for the sensitivity of heat-health outcomes to a wider suite of environmental factors than those typically considered.

## Supplementary Information

Below is the link to the electronic supplementary material.

Supplementary file1 (DOCX 172 KB)
