# Supplementary material for: Impact of environmental factors on heat-associated mortalities in an urban desert region
Source: Int J Biometeorol. 2022 Sep 10;66(10):2133–46. doi: 10.1007/s00484-022-02346-7 (PMC9463968; doi:10.1007/s00484-022-02346-7)
Supplement: Supplementary file 1 — Supplementary file1 (DOCX 172 KB) [file 484_2022_2346_MOESM1_ESM.docx]

**SUPPLEMENTARY INFORMATION**

**Table S1.** Summary of model formulations using a data withhold and test approach for each of the ten subsets of data described in the text. The testing rounds correspond to those listed in Table 1 and indicate the environmental variables included as potential predictors. Models were fit using all data excluding the subset of data listed. The best-performing model chosen in each round of formulations was selected based on the lowest mean absolute error (MAE) between the model predictions (rounded to the nearest whole number) and observed number of heat-associated mortalities for the omitted subset of data. All predictors included in the best-performing model for each scenario were required to exhibit statistical significance (p<0.001). In some scenarios there were multiple models that exhibited the same lowest MAE; these cases are indicated in the table as “multiple formulas.” Note that the order of variables listed in each formula is arbitrary.

| Omitted Subset of Data | Testing Round | Best Performing Model Formula | Lowest MAE |
| --- | --- | --- | --- |
| 1 | 1 | Year + Daily Average Air Temperature | 0.595 |
| 1 | 2 | Year + Day of Year + Daily Average Air Temperature + Average of Previous 4 Days Daily Average Air Temperature | 0.542 |
| 1 | 3 | Day of Year + Daily Average Air Temperature + Average of Previous 5 Days Daily Average Air Temperature + Average of Previous 5 Days Daily Average Dew Point Temperature | 0.503 |
| 1 | 4 | Day of Year + Daily Average Air Temperature + Average of Previous 5 Days Daily Average Air Temperature + Average of Previous 5 Days Daily Average Dew Point Temperature | 0.503 |
| 1 | 5 | Day of Year + Daily Average Air Temperature + Average of Previous 5 Days Daily Average Air Temperature + Average of Previous 5 Days Daily Average Dew Point Temperature | 0.503 |
| 1 | 6 | Multiple Formulas | 0.503 |
| 1 | 7 | Day of Year + Daily Average Air Temperature + Previous Day Daily Average PM2.5 + Average of Previous 3 Days Daily Average Air Temperature + Average of Previous 4 Days Daily Average Dew Point Temperature + Average of Previous 5 Days Daily Average PM10 | 0.497 |
| 2 | 1 | Daily Average Air Temperature | 0.614 |
| 2 | 2 | Multiple Formulas | 0.575 |
| 2 | 3 | Multiple Formulas | 0.569 |
| 2 | 4 | Multiple Formulas | 0.542 |
| 2 | 5 | Multiple Formulas | 0.536 |
| 2 | 6 | Year + Day of Year + Previous Day Daily Average Air Temperature + Average of Previous 2 Days Daily Average Dew Point Temperature + Average of Previous 2 Days Daily Average PM10 | 0.556 |
| 2 | 7 | Multiple Formulas | 0.536 |
| 3 | 1 | Year + Daily Average Air Temperature | 0.614 |
| 3 | 2 | Year + Daily Average Air Temperature | 0.614 |
| 3 | 3 | Multiple Formulas | 0.582 |
| 3 | 4 | Multiple Formulas | 0.582 |
| 3 | 5 | Multiple Formulas | 0.582 |
| 3 | 6 | Multiple Formulas | 0.582 |
| 3 | 7 | Multiple Formulas | 0.582 |
| 4 | 1 | Year + Daily Average Air Temperature | 0.743 |
| 4 | 2 | Year + Day of Year + Average of Previous 4 Days Daily Average Air Temperature | 0.618 |
| 4 | 3 | Year + Day of Year + Average of Previous 4 Days Daily Average Dew Point Temperature + Average of Previous 5 Days Daily Average Air Temperature | 0.612 |
| 4 | 4 | Multiple Formulas | 0.612 |
| 4 | 5 | Year + Day of Year + Average of Previous 4 Days Daily Average Dew Point Temperature + Average of Previous 5 Days Daily Average Air Temperature | 0.612 |
| 4 | 6 | Year + Day of Year + Daily Average PM10 + Average of Previous 2 Days Daily Average Dew Point Temperature + Average of Previous 5 Days Daily Average Air Temperature | 0.605 |
| 4 | 7 | Year + Day of Year + Daily Average PM10 + Average of Previous 2 Days Daily Average Dew Point Temperature + Average of Previous 5 Days Daily Average Air Temperature | 0.605 |
| 5 | 1 | Year + Daily Average Air Temperature | 0.586 |
| 5 | 2 | Multiple Formulas | 0.533 |
| 5 | 3 | Multiple Formulas | 0.493 |
| 5 | 4 | Multiple Formulas | 0.493 |
| 5 | 5 | Year + Day of Year + Daily Average Air Temperature + Daily Average PM2.5 + Average of Previous 3 Days Daily Average Air Temperature + Average of Previous 5 Days Daily Average Dew Point Temperature | 0.474 |
| 5 | 6 | Multiple Formulas | 0.493 |
| 5 | 7 | Year + Day of Year + Daily Average Air Temperature + Daily Average PM2.5 + Average of Previous 3 Days Daily Average Air Temperature + Average of Previous 5 Days Daily Average Dew Point Temperature | 0.474 |
| 6 | 1 | Year + Daily Average Air Temperature | 0.592 |
| 6 | 2 | Year + Daily Average Air Temperature + Previous Day Daily Average Air Temperature | 0.526 |
| 6 | 3 | Year + Day of Year + Daily Average Air Temperature + Previous Day Daily Average Air Temperature + Average of Previous 4 Days Daily Average Dew Point Temperature | 0.493 |
| 6 | 4 | Year + Day of Year + Daily Average Air Temperature + Previous Day Daily Average Air Temperature + Average of Previous 4 Days Daily Average Dew Point Temperature | 0.493 |
| 6 | 5 | Year + Day of Year + Daily Average Dew Point Temperature + Previous Day Daily Average Air Temperature + Average of Previous 5 Days Daily Average PM2.5 | 0.461 |
| 6 | 6 | Multiple Formulas | 0.493 |
| 6 | 7 | Year + Day of Year + Daily Average Dew Point Temperature + Previous Day Daily Average Air Temperature + Average of Previous 5 Days Daily Average PM2.5 | 0.461 |
| 7 | 1 | Year + Daily Average Air Temperature | 0.579 |
| 7 | 2 | Year + Daily Average Air Temperature + Average of Previous 2 Days Daily Average Air Temperature | 0.520 |
| 7 | 3 | Year + Day of Year + Average of Previous 2 Days Daily Average Air Temperature + Average of Previous 3 Days Daily Average Dew Point Temperature | 0.474 |
| 7 | 4 | Year + Day of Year + Previous Day Daily Maximum 8hr Average O3 + Average of Previous 2 Days Daily Average Air Temperature + Average of Previous 3 Days Daily Average Dew Point Temperature | 0.467 |
| 7 | 5 | Multiple Formulas | 0.467 |
| 7 | 6 | Year + Day of Year + Average of Previous 2 Days Daily Average Air Temperature + Average of Previous 3 Days Daily Average Dew Point Temperature | 0.474 |
| 7 | 7 | Multiple Formulas | 0.467 |
| 8 | 1 | Year + Daily Average Air Temperature | 0.691 |
| 8 | 2 | Day of Year + Daily Average Air Temperature + Average of Previous 5 Days Daily Average Air Temperature | 0.638 |
| 8 | 3 | Year + Day of Year + Average of Previous 3 Days Daily Average Air Temperature + Average of Previous 3 Days Daily Average Dew Point Temperature | 0.612 |
| 8 | 4 | Year + Day of Year + Average of Previous 3 Days Daily Average Air Temperature + Average of Previous 3 Days Daily Average Dew Point Temperature | 0.612 |
| 8 | 5 | Year + Day of Year + Average of Previous 2 Days Daily Average Air Temperature + Average of Previous 4 Days Daily Average Dew Point Temperature + Average of Previous 5 Days Daily Average PM2.5 | 0.586 |
| 8 | 6 | Year + Day of Year + Average of Previous 2 Days Daily Average Air Temperature + Average of Previous 4 Days Daily Average Dew Point Temperature + Average of Previous 5 Days Daily Average PM10 | 0.586 |
| 8 | 7 | Day of Year + Daily Average Air Temperature + Average of Previous 2 Days Daily Average Air Temperature + Average of Previous 5 Days Daily Average Dew Point Temperature + Average of Previous 5 Days Daily Average PM2.5 + Average of Previous 5 Days Daily Average PM10 | 0.579 |
| 9 | 1 | Year + Daily Average Air Temperature | 0.599 |
| 9 | 2 | Year + Day of Year + Daily Average Air Temperature + Average of Previous 5 Days Daily Average Air Temperature | 0.513 |
| 9 | 3 | Multiple Formulas | 0.513 |
| 9 | 4 | Multiple Formulas | 0.513 |
| 9 | 5 | Multiple Formulas | 0.513 |
| 9 | 6 | Multiple Formulas | 0.513 |
| 9 | 7 | Day of Year + Daily Average Air Temperature + Previous Day Daily Average PM2.5 + Average of Previous 2 Days Daily Average Dew Point Temperature + Average of Previous 2 Days Daily Average PM10 + Average of Previous 5 Days Daily Average Air Temperature | 0.507 |
| 10 | 1 | Year + Daily Average Air Temperature | 0.599 |
| 10 | 2 | Multiple Formulas | 0.539 |
| 10 | 3 | Year + Day of Year + Daily Average Air Temperature + Daily Average Dew Point Temperature + Average of Previous 3 Days Daily Average Air Temperature | 0.526 |
| 10 | 4 | Year + Day of Year + Daily Average Air Temperature + Daily Average Dew Point Temperature + Average of Previous 3 Days Daily Average Air Temperature | 0.526 |
| 10 | 5 | Year + Day of Year + Daily Average Air Temperature + Daily Average Dew Point Temperature + Daily Average PM2.5 | 0.520 |
| 10 | 6 | Multiple Formulas | 0.520 |
| 10 | 7 | Year + Daily Average Air Temperature + Daily Average PM10 + Average of Previous 5 Days Daily Average PM2.5 | 0.513 |

**Table S2.** Measure of collinearity between predictor variables in the final best-performing model. Values presented are the Pearson correlation coefficient (R) for linear correlations between variables. Note that italicized values are not significant at p<0.01.

|  | Daily average air temperature | Average of previous 5 days daily average air temperature | Year | Day of year | Average of previous 5 days daily average dew point temperature | Average of previous 5 days 24-hr average PM2.5 |
| --- | --- | --- | --- | --- | --- | --- |
| Average of previous 5 days daily average air temperature | 0.785 |  |  |  |  |  |
| Year | *-0.014* | *-0.007* |  |  |  |  |
| Day of year | 0.359 | 0.474 | *-0.002* |  |  |  |
| Average of previous 5 days daily average dew point temperature | 0.403 | 0.514 | 0.078 | 0.703 |  |  |
| Average of previous 5 days 24-hr average PM2.5 | 0.123 | 0.182 | -0.420 | -0.116 | -0.125 |  |
| 24-hr average PM10 | 0.155 | 0.150 | -0.090 | *-0.012* | -0.115 | 0.267 |

**
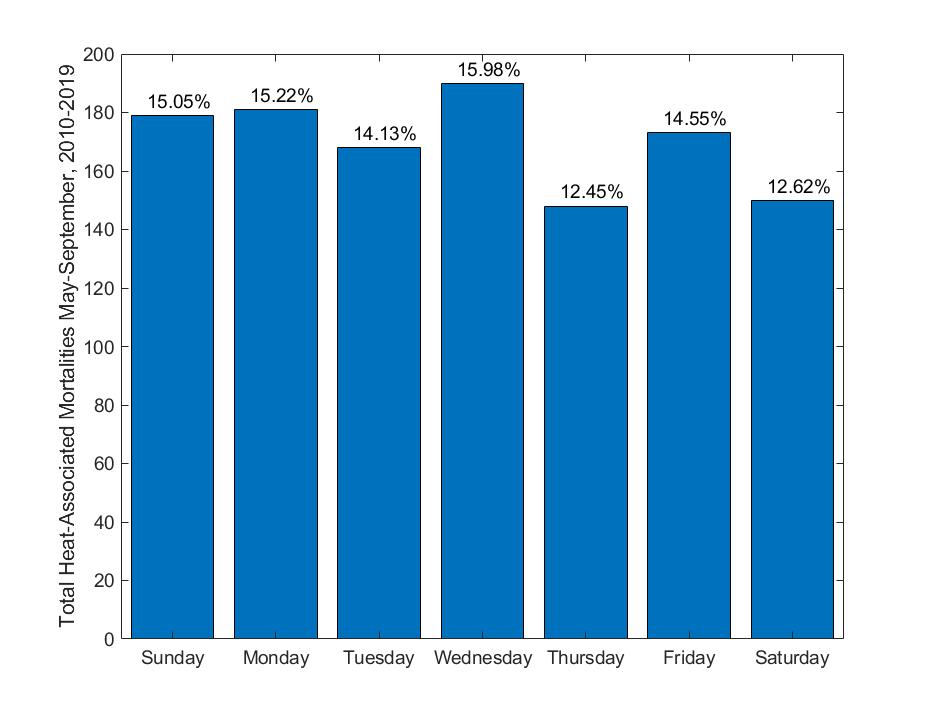
**

**Figure S1.** Heat-associated mortalities in Maricopa County May-September, 2010-2019 by day of week.

**
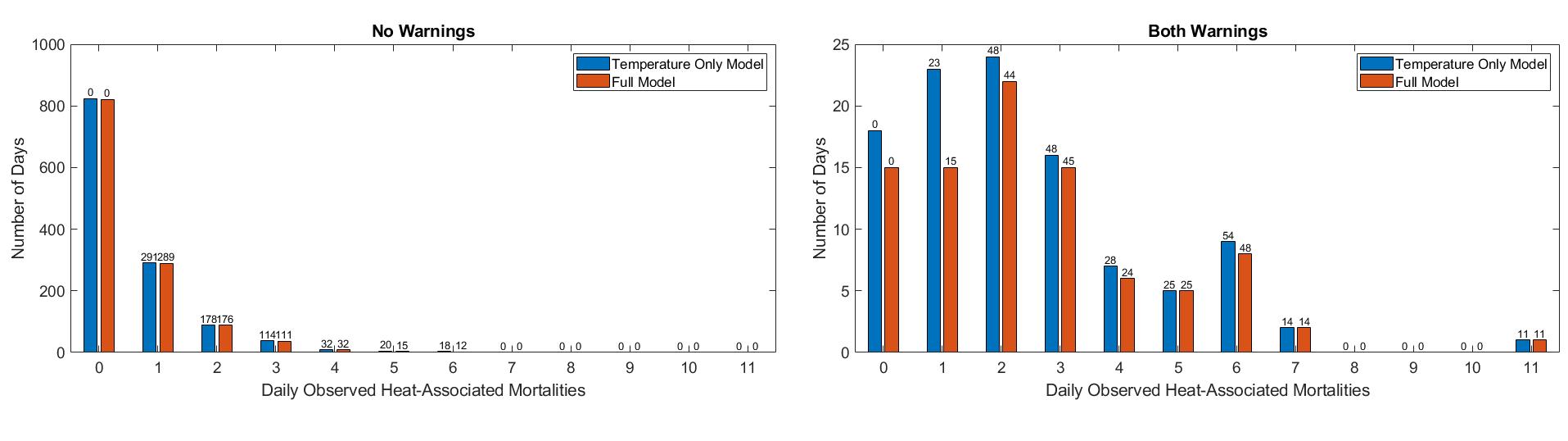
**

**Figure S2.** Comparison of days with no heat warning and no modeled warning (first plot) and days with both a heat warning and a modeled warning (second plot). Bars indicate the number of days falling into each category, while the numbers at the top of the bars indicate the number of heat-associated moralities in the category. Blue bars represent the temperature only model, while orange bars are for the full model.
